# Association between the Vicious Cycle of Diabetes-Associated Complications and Glycemic Control among the Elderly: A Systematic Review

**DOI:** 10.3390/medicina54050073

**Published:** 2018-10-15

**Authors:** Muhammad Atif, Quratulain Saleem, Zaheer-Ud-Din Babar, Shane Scahill

**Affiliations:** 1 Department of Pharmacy, The Islamia University of Bahawalpur, Bahawalpur 63100, Pakistan; q.pharma@gmail.com; 2 Department of Pharmacy, University of Huddersfield, Huddersfield HD6 2LZ, UK; Z.Babar@hud.ac.uk; 3 School of Management, Massey University, Auckland 0632, New Zealand; s.scahill@massey.ac.nz

**Keywords:** health related quality of life, diabetes, glycemic control, depression, cognition, frailty, malnutrition, physical functioning, pain, self-care, healthcare professionals

## Abstract

*Background and objectives*: Improved quality of life (QoL) and life expectancy of elderly diabetic patients revolves around optimal glycemic control. Inadequate glycemic control may lead to the development of diabetes-associated complications (DAC), which not only complicate the disease, but also affect morbidity and mortality. Based on the available literature, the aim was to elucidate the vicious cycle underpinning the relationship between diabetes complications and glycemic control. *Materials and Methods*: A comprehensive literature search was performed to find eligible studies published between 1 January 2000 and 22 September 2018 pertaining to diabetes complications and glycemic control. *Results*: Initially, 261 studies were retrieved. Out of these, 67 were duplicates and therefore were excluded. From the 194 remaining articles, 85 were removed based on irrelevant titles and/or abstracts. Subsequently, the texts of 109 articles were read in full and 71 studies were removed at this stage for failing to provide relevant information. Finally, 38 articles were selected for this review. Depression, impaired cognition, poor physical functioning, frailty, malnutrition, chronic pain, and poor self-care behavior were identified as the major diabetes-associated complications that were associated with poor glycemic control in elderly diabetic patients. *Conclusions*: This paper proposes that diabetes-associated complications are interrelated, and that impaired glycemic control aggravates diabetes complications; as a result, patient’s self-care abilities are compromised. A schema is generated to reflect a synthesis of the literature found through the systematic review process. This not only affects patients’ therapeutic goals, but may also hamper their health-related quality of life (HRQoL) and financial status.

## 1. Introduction

Diabetes is a leading cause of multiple morbidities in the elderly population, which reduces their quality of life and life expectancy. With an estimated global prevalence of 9% among adults, diabetes is expected to be the seventh preeminent cause of death by 2030 [1]. The elderly are the major victims of diabetes specifically, type-2 diabetes mellitus (T2DM). Around 30% of people in the world aged between 65 and 85 years are afflicted by T2DM; including 11.2 million Americans [2]. Similarly, the high prevalence of the disease is found among older people (70 to 79 years) living in Europe, North America and Australia [3].

The elderly are prone to various physical and mental problems due to the natural aging process, and multiple ailments associated with diabetes make the aging process even more difficult and cumbersome [4]. Depression, impaired cognition, poor physical functioning (PF), frailty, malnutrition, chronic pain, and poor self-care behaviors are the major issues associated with diabetes in the elderly [5,6,7,8]. These diabetes-associated complications (DAC) may directly affect a patient’s health-related quality of life (HRQoL), posing severe economic burden on the patient and society at large. It is a grim reality that despite having strong negative associations with clinical outcomes and patient’s HRQoL, DAC are overlooked by healthcare professionals when managing patients with T2DM. Clinicians appear to focus more on providing conventional treatment regimens, ignoring patient’s additional needs that are related to DAC.

Recent guidelines [9] for the management of diabetes in the elderly, provided by the International Diabetes Federation (IDF), have stressed that thorough patient examination is required in-order to evaluate the presence of ailments considered to be DAC. It has been further emphasized that, in addition to providing conventional clinical care to elderly diabetic patients, these DAC should be adequately managed to improve patients’ overall health [9]. The purpose of this systematic literature review is to describe the association between DAC and glycemic control among elderly patients with diabetes and highlight the implications for patient health outcomes. Based on the available literature, a vicious cycle describing the relationship between DAC and glycemic control is proposed, and healthcare professionals are urged to optimize the management of elderly with diabetes the consideration of this cycle.

## 2. Methods

We systematically identified studies related to diabetes and DAC, published in the scientific literature during the period from 1 January 2000 to 22 September 2018. Inclusion and exclusion criteria for the studies are outlined in Table 1. We followed the PRISMA (Preferred Reporting Items for Systematic Reviews and Meta-Analysis) guidelines [10] in the preparation of this review. We have developed a protocol of methods, which can be assessed at http://www.crd.york.ac.uk/PROSPERO/display_record.asp?ID=CRD42016030172.

### 2.1. Search Methods

A comprehensive literature search was conducted using Google Scholar, Medline, PubMed, Scopus, SpringerLink, and ScienceDirect databases. “Diabetes”, “Diabetes mellitus”, “Type 1 diabetes mellitus”, “Type 2 diabetes mellitus”, “Glycemic control”, “Depression”, “Cognition”, “Frailty”, “Malnutrition”, “Physical functioning”, “Pain”, “Self-care”, and “Healthcare professionals” were used as keywords in diverse combinations with Boolean and Medical Subject Headings (MeSH) searches to identify all relevant studies.

Further publications were identified by manual searching of the references of related papers and review articles. Various journals in the diabetes and endocrinology domain were searched to identify further relevant articles.

### 2.2. Data Extraction (Selection and Coding)

A data extraction form was developed. The items on the data extraction form were finalized after discussion amongst members of the research team. The extracted data included the first author’s name, year of data (the midpoint of the study’s time period), study design, study setting, data collection method, characteristics of the patients (sample size and age), and major outcomes.

Retrieved articles were imported into Endnote X7 to remove duplicates, and they were included or excluded according to the predefined criteria. QS and MA independently assessed the titles and abstracts to select the studies. After preliminary screening, a full-text assessment was made to determine the final inclusion of articles for this review. Disagreement amongst the research team regarding the eligibility of any study was resolved through discussion and mutual agreement in the research team meetings. All authors agreed with the final studies selected for the review. Two independent reviewers checked all studies to verify the validity of the screening procedure.

### 2.3. Risk of Bias (Quality) Assessment

Two independent reviewers evaluated the data. The data were analyzed based on the quality of the data [11]. Any disagreements raised among the reviewers were resolved through discussion in the research team meeting.

### 2.4. Strategy for Data Synthesis

A systematic review was undertaken to ensure that synthesis produced was sourced from the maximum possible complete collection of relevant literature.

## 3. Results

**Figure 1:**
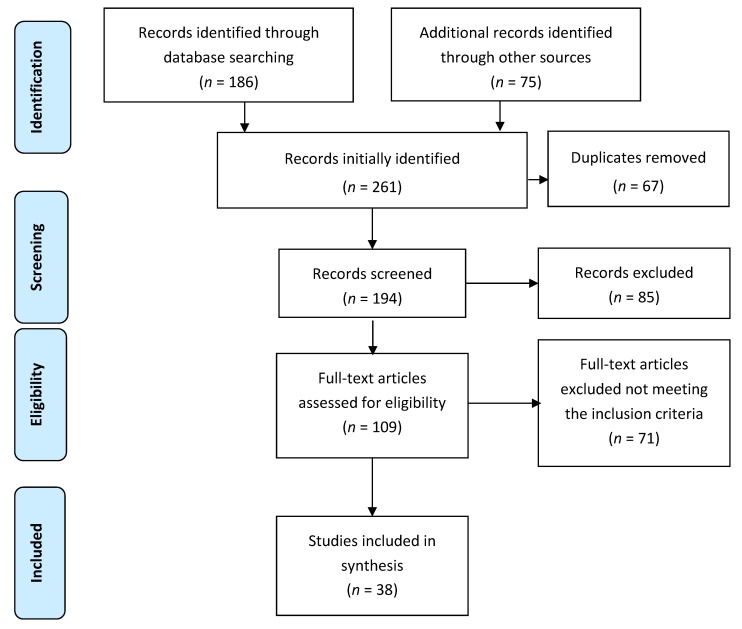
Schematic diagram explaining the assortment of studies/reports (2009 PRISMA flow diagram).

Initially, 261 studies were retrieved. Out of these, 67 were duplicates, and therefore they were excluded. From the 194 remaining articles, 85 were removed based as irrelevant titles and/or abstracts. Subsequently, the texts of 109 articles were read in full, and 71 studies were removed at this stage for failing to provide relevant information. Finally, 38 articles were selected for this review (Figure 1).

### 3.1. Characteristics of Selected Studies

The major characteristics of the 38 studies meeting the criteria for review are described in Table 2. Nineteen studies were conducted in the United States (US) [6,8,12,13,14,15,16,17,18,19,20,21,22,23,24,25,26,27,28] four in the United Kingdom (UK) [29,30,31,32], three in China [7,33,34], three in Canada [35,36,37], two in Turkey [38,39], and one in each of the Netherlands [40], Switzerland [41], Taiwan [42], Mauritius [43], Malaysia [44], Finland [45], and Pakistan [46]. Sixteen studies utilized cross-sectional [7,8,13,23,24,26,32,33,34,37,38,39,43,44,45,46] study designs, three were case-control studies [29,30,31] and 15 were longitudinal or prospective studies [6,14,15,16,17,20,22,25,27,28,35,36,40,41,42]. The remaining studies adopted mixed study designs (cross-sectional and longitudinal) [12,18,19,20,21]. There was significant variation in the sample size of the included studies, ranging from 60 [13] to 9249 participants [25]. In one case-control study, only 35 cases and 35 controls were included [29]. Only one study specifically dealt with both type 1 diabetes mellitus (T1DM) and T2DM patients [8]. Fourteen studies were related specifically to T2DM [6,21,23,28,32,33,34,36,37,39,42,43,44,46]. Only one study described the association between hyperglycemia and cognitive decline [14]. The remainder of the studies did not specify the type of diabetes. In most of the studies, all of the participants were elderly people with diabetes. In a few studies however, the participants were aged greater than 18 years, but included elderly patients (>60 years) as a sub-group. Therefore, the mean age was utilized for the analysis of these studies [8,21,23,28,32,36,39,43,44].

### 3.2. Study Evaluation Criteria

The studies were evaluated using a standard quality testing protocol developed by Kamet *et al.* [11]. The studies included in the review were tested and scored on the basis of 14 items (each item with a maximum score of 2). Three types of scores may be assigned to each item. The item was scored as 2 if the standard criteria were met, and 1 or 0 if the quality criteria were either partially met or not met at all. If a specific item did not match the nature of the study, it was not scored, and the item(s) were excluded from the summary scores. The percentage of scores for each study was calculated which indicated the quality of the study in numerical form. The quality scores of the majority of studies ranged between 80% and 100%, and nine studies had a maximum score of 100% [7,12,24,25,27,32,34,36,43]. Four studies scored between 70% and 80% [23,28,29,39], and one study scored below 70%, but it was above the inclusion criteria requirement of 66% [41]. Detailed quality scoring of the studies is provided in Table 3.

## 4. Discussion

### 4.1. Interrelationship between Diabetes-Associated Complications and Clinical Outcomes

#### 4.1.1. Depression

Diabetes directly affects mood levels, and depression is the basic clinical manifestation of significantly altered mood. Diabetes-related hyperglycemia and hypoglycemia are the primary causes of depression in elderly diabetic patients [8,46]. Studies have illustrated that there is a high prevalence of depression among elderly diabetic patients [12,27]. Depressed diabetic patients are more prone to other health-related problems, for instance: malnutrition, poor cognition, disability, and tendency for falls [4]. Similarly, diabetic patients can demonstrate low self-esteem as well as motivational problems for achieving good glycemic control. Consequently, they show poor adherence to therapy [5,6,8]. Studies have also revealed that depressed elderly patients show minimum interest in self-care behavior [23,47] and thus, their glycemic targets remain hard to achieve [38]. Supportive behavior of healthcare professionals and family members can play a vital role in tackling depression in elderly diabetic patients. Prescribing antidepressants and efficient management of glycemic levels can also help to ameliorate depression in these patients.

#### 4.1.2. Impaired Cognition

Cognitive decline is a component of the normal aging process, but diabetes accelerates cognitive decline in the elderly [14,16]. This is due to a reduction of extracellular glucose levels in the hippocampus, which limits activity in memory processing [5]. Studies have supported the fact that the risk of vascular dementia (1.3–3.4 folds) and Alzheimer’s disease increases with diabetes [2,4]. Prolonged and intensive insulin therapy in elderly diabetes patients has been shown to increase the risk of hypoglycemia, which has a direct association with impaired cognitive function [15]. Compromised cognition further aggravates hypoglycemia by making the patient less aware of hypoglycemic symptoms [15]. A forgetful patient also becomes unable to recognize the importance of glycemic control and self-management of their diabetes. The result is that patients suffer from poor glycemic condition which continues to afflict them as the disease progresses [13,31].

A timely diagnosis and prescribing medicines which decrease the risk of developing cerebrovascular anomalies may help prevent the expected cognitive dysfunction over time in these patients. Moreover, improving the glycemic control with pharmacotherapy may also help avert the transformation from mild cognitive decline to severe dementia in this cohort [48,49]. Healthcare professionals need to devise ways to increase concordance with prescribed medication regimens. Patients’ families and caregivers can also play a major role in supporting the diabetic patient to be more adherent of their treatment regimen, and thereby achieving positive disease outcomes.

### 4.2. Poor Physical Functioning

Low levels of physical functioning (LPF) and mobility are associated with the aging process because of reduced bone strength, muscle tone, and elasticity [50]. An association between LPF and diabetes has been established [7,22]. Functional decline is seen in most elderly people with diabetes who have elevated HbA1c levels [19]. Such patients show impaired activities of daily living (ADL) and instrumental activities of daily living (IADL) [7]. Poor PF may reduce self-care in patients which can adversely affect the self-management of diabetes and may result in poorer health outcomes [4,22]. Providing physiotherapy and functional aids to patients can improve their self-management by enabling them to adhere to a healthy diet plan, regular blood glucose monitoring, exercising, and foot care with ongoing monitoring [33].

#### 4.2.1. Frailty

Hypoglycemia-induced falls are very common in elderly diabetic patients [18]. Studies have shown that the elderly diabetic patient has a greater risk of falling than any other group of older adults [17,25]. Correlation between frailty and glycemic control has been shown in a study where 77% of patients with HbA1c levels ≤7 had a fall [18]. This reveals that poor glycemic control not only increases the chances of falls in frail diabetic patients but also in non-frail elderly diabetic patients [40]. Frailty in diabetes patients is also associated with reduced ADL. Moreover, impaired cognition and depression that are associated with poor glycemic control also contribute to frailty in these patients [47]. Similarly, a direct association between frailty, poor self-efficacy, and inadequate self-care has also been established in elderly diabetes patients [51]. These associations can further aggravate the disease, and the patients suffer in terms of morbidity and mortality. Improving self-care activities and managing hypoglycemia can prevent the probability of falls occurring in these patients [35]. These patients must be counseled about keeping immediate-acting sugar substitutes on-hand in order to treat hypoglycemia in an emergency situation.

#### 4.2.2. Pain

The prevalence of pain is high in elderly diabetic patients [24]. Diabetes is a major determinant of axonal and sensory neuropathy, which is expected to develop in half of diabetic patients who have suffered from the disease for two decades or more [26]. Neuropathy causes intense pain in some patients in the peripheral areas of the body [52,53]. A study has revealed a 26.4% prevalence of painful diabetes-related peripheral neuropathy, affecting their quality of life in many ways [32]. Another study has shown the significant impact of pain associated with diabetes-related neuropathy, affecting patients’ physical and mental wellbeing [26]. Consistent hyperglycemia associated with diabetes is the major reason for the development of neuropathy, which has been evidenced by studies that have shown that hyperglycemia and increased levels of HbA1c are observed in patients with neuropathy [18,26]. Additionally, diabetic patients also feel pain due to skin and soft tissue infections [54]. Pain reduces PF and self-care activities [55], such that patients who feel persistent pain are unable to perform much needed tasks, for example, glucose monitoring or following a diet plan. Prescribing analgesics and educating patients that they need to keep blood glucose levels within the normal range can be beneficial in preventing development of painful neuropathy and skin infections [52]. Adopting self-care activities relating to foot care is also important in this regard.

### 4.3. Malnutrition

Malnutrition is another significant and debilitating issue that elderly people are faced with. About 5–10% of independently living and 30–60% of hospitalized elderly patients are malnourished [38]. Malnutrition diminishes the health and well-being of diabetic patients, and affects their PF as well. The association between diabetes and the high prevalence of malnutrition in elderly diabetes patients is well established [29,41]. In a study it was found that nearly one quarter (23.7%) of diabetic patients were malnourished, despite having elevated plasma glucose levels, probably because of being in a hypermetabolic state [38]. Besides, protein catabolism and weight loss seen in elderly patients with diabetes is another reason for being malnourished. Depression and poor IADL also result in malnutrition in a way that depressed patients lack appetite, which consequently leads to poor nutritional status [29]. Irrespective of the reason, malnutrition is a risk factor for hypoglycemia that may further worsen physical and mental wellbeing [3], and thereby negatively affect levels of self-care. Consequently, poor self-care may lead to poor glycemic control and poor clinical outcomes [33]. A balanced diet containing optimum levels of essential nutrients is necessary for elderly diabetic patients. Healthcare practitioners can prescribe supplements and provide a balanced diet plan to help prevent malnutrition. Improving physical and mental health and self-care activities can also improve the diabetic patient’s nutritional status by assisting the patient to plan, prepare, and eat a balanced diet.

### 4.4. Poor Self-Care

Self-care is described as all the activities performed by a diabetic patient to adhere to a prescribed diet plan, and a drug therapy regime to achieve standard glycemic targets to effectively manage diabetes [56]. The extent to which patients undertake self-care has a direct relationship with glycemic control. Studies demonstrate that effective self-care translates into better glycemic control, leading to improved health outcomes and vice versa [23,33]. DAC also has links with self-care, as depression, impaired cognition, poor PF, frailty, malnutrition, and pain may hamper the ability to undertake self-care in diabetic patients, due to the associated physical and mental discomfort. Consequently, the patient cannot keep pace with self-management of the disease because of poor self-care, and may suffer from worse clinical outcomes [8,13,24,41]. The best way to improve self-care abilities and to achieve adequate glycemic control is through the prevention of DAC in the first place. In this regard, there is a need for the early detection and management of DAC to boost the level of self-care among elderly diabetic patients.

### 4.5. A Schema of the Vicious Cycle of Diabetes-Associated Complications (DAC) and Their Outcomes

**Figure 2:**
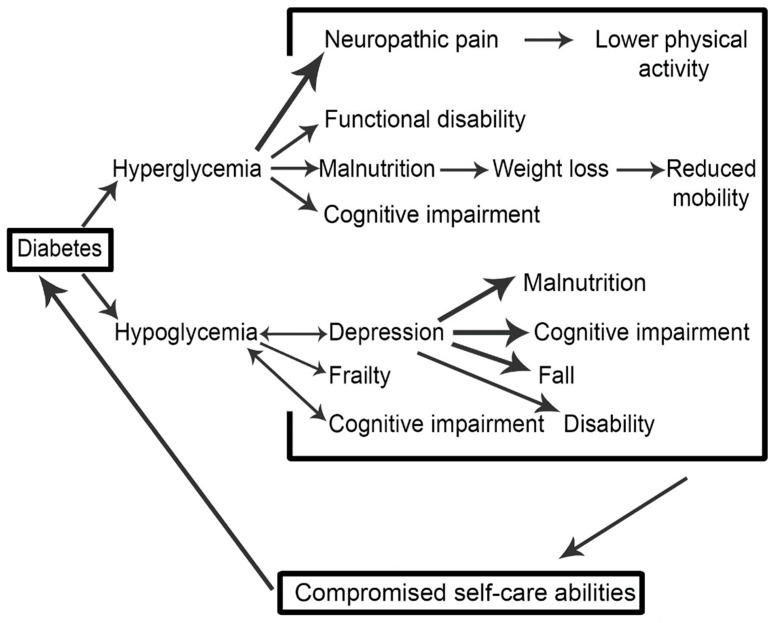
A vicious cycle explaining the relationship between of diabetes associated complications and glycemic control.

Figure 2 is a schematic interpretation of our synthesis of the literature associated with diabetes-related complications in the older adult. The schema demonstrates in a visual form the association of DAC with glycemic control, and how these linkages are connected to self-care abilities, potentially forming a vicious cycle. Impaired glycemic control (hyperglycemia or hypoglycemia) leads to the development of DAC that again results in poor glycemic control. If it continues, such a mechanism may result in worse health outcomes, and subsequent high societal burden. The aim of developing this schema is to pictorially represent the complexity associated with diabetes management, and to draw the literature together around diabetes-associated complications (DAC) whilst thinking about how the relationships between the various complications might manifest. Of course, outcomes for patients and wider society are ultimately thought about and implicated.

Diabetes complications (microvascular and macrovascular) are not considered in the proposed vicious cycle, despite the fact that these complications can also affect glycemic control. It is suggested that future studies should attempt to address this complex paradigm. Another limitation of this systematic review is that only the elderly group of diabetic patients was considered. We included this subgroup of diabetes patients because this is the most vulnerable group for the development of DAC, as compared to other age groups. The association between DAC and glycemic control needs to be better understood through modelling the literature within this context. Future studies should also investigate this relationship empirically, based on patient characteristics such as gender, education level etc.

## 5. Conclusions

A thorough review of literature has revealed that DACs that are associated with diabetes is a complex area, and in the elderly the literature can be formulated into a schema demonstrating its interconnectedness. Impaired glycemic control aggravates DAC, and as a result, patients’ self-care abilities are compromised. This not only affects patients’ therapeutic goals, but also their HRQoL.

Improved HRQoL and increased life expectancy are major health intentions for elderly diabetic patients, which can be achieved by setting glycemic targets and maintaining good glycemic control. This calls for a collaborative effort by healthcare professionals (physicians, nurses, and pharmacists), patients’ families and caregivers, and the patients themselves. All efforts performed by the caregivers and the healthcare professionals may fail if patients show a reluctance to perform self-care activities to manage the disease. Healthcare professionals should consider the management of DAC while making treatment decisions, rather than simply following conventional drug therapy strategies. Prioritizing DAC can not only help to break the vicious cycle, but it can also help to improve patients’ HRQoL.

## Figures and Tables

**Table 1:** Inclusion and exclusion criteria.

Sr. No.	Inclusion Criteria
1	Studies on diabetes-associated complications published during the period from 1 January 2000 to 22 September 2018.
2	All original research articles describing the association between diabetes, diabetes-associated complications, and glycemic control in the elderly, available in the scientific literature.
3	Studies conducted in elderly (≥60 years) diabetic patients.
4	Studies having quality evaluation scores of >66%.
	**Exclusion criteria**
1	Studies published in a language other than English.
2	Studies without clear inclusion and exclusion criteria.
3	Studies without clearly stated outcomes.

**Table 2:** Study characteristics.

(First Author) (Year) (Country)	Main Objective	Design	Setting	Type of Diabetes	Sample Demographics	Main Results
Blazer, D.G. (1986–1997) (US) [12]	Assessment of association between depression, obesity and diabetes.	Observational, cross-sectional and longitudinal survey	House hold survey	Not specified	*N* = 4162 Age ≥ 65 years	In the controlled and uncontrolled analyses, functional impairment (*p* < 0.001), female gender (*p* < 0.05), cognitive impairment (*p* < 0.01), and lower education were found to be associated with depression, diabetes, and high BMI (*p* < 0.05). The frequency of comorbidity between depression and diabetes was 2.6%.
Black, S.A. (1995–2001) (US) [6]	Assessment of impact of diabetes and depression on poor health outcomes in diabetes patients.	Longitudinal survey	In-home face-to-face interviews	T2DM	*N* = 2830 Age ≥65 years	Significant relationship was seen between depression and diabetes. About 24% of the patients had minor depression, 9% of the patients had major depression, and 47% of the patients had diabetes with minimum levels of depression.
Chiechanowski, P.S. (1999) (US) [8]	Assessment of association between diabetes, depression, PF, self-care, and HbA1c levels. Moreover, assessment of intensity of depression and HbA1c levels in patients with T1DM as compared the patients with T2DM.	Cross-sectional observational study	Tertiary care specialty clinic	T1DM, T2DM	*N* = 276 T1DM patients *N* = 199 T2DM patients Mean age of the relevant group = 48.8 ± 15.9 years	A significant association was seen between depression, glycemic control (*p* < 0.0001), HbA1c levels (*p* < 0.0001), PF (*p* < 0.01), and adherence to self-care behavior (*p* < 0.0001). Similarly, a significantly greater number (66.7%) of T1DM patients with HbA1c levels >8 were found to be depressed than T2DM depressed patients (37.5%) (*p* = 0.02).
Zuberi, S.I. (2008–2009) (Pakistan) [46]	Assessment of association between depression, self-care, and diabetes.	Cross-sectional study	Tertiary care hospital	T2DM	*N* = 286 diabetes patients Age = 31–60 years	Depression in male diabetes patients was lesser than female diabetes patients by the values; 39.2 and 60.8 respectively (*p* = 0.03). Moreover, HbA1c levels were significantly higher in depressed patients than in non-depressed diabetes patients (8.5% vs. 7.7%, *p* < 0.001).
Munshi, M. (2005) (US) [13]	Assessment of the association between cognitive dysfunction and glycemic control.	Cross-sectional study	Geriatric diabetic clinic	Not specified	*N* = 60 Age ≥ 70 years	Results showed that 34% of diabetes patients had low scores of CIB, whereas 38% of the patients had low CDT scores. Both the tests CIB (r = −0.37, *p* < 0.004) and CDT (r = −0.38, *p* < 0.004) had an inverse correlation with HbA1c levels. Furthermore, 33% of the patients were depressed, and 33% of the patients had history of falls, whereas 39% of the patients had poor IADL scores.
Yaffe, K.Y. (1998–1999) (US) [14]	To investigate the association between metabolic syndrome and cognitive function, and effect of inflammation on this association.	Longitudinal cohort study	Sacramento area and the surrounding California counties	Hyperglycemia associated with metabolic syndrome	*N* = 1624 Age ≥ 60 years	Rate of cognitive decline was found to be greater in patients with metabolic syndrome having hyperglycemia. Low scores of DelRec (*p* = 0.02) proved the finding. Similarly, low 3MS scores (*p* = 0.03) in the patients with inflammation, showed the impact of inflammation on cognitive decline.
Yaffe, K. (1997–2006) (US) [16]	Association between diabetes and cognitive decline and impact of glycemic control on cognitive function.	Prospective cohort study	Community clinics	Not specified	*N* = 3069 Age = 70–79 years	Participants with DM showed decline in cognitive function, and had low scores of cognitive status, i.e., 3MS (*p* = 0.001) and DSS (*p* = 0.001). Likewise, a significant association was also observed between HbA1c levels and cognitive decline, which was shown by low 3MS (*p* = 0.003) and DSS (*p* = 0.04) scores in the diabetes patients.
Yaffe, K. (1997–2008) (US) [15]	Assessment of association between hypoglycemia and dementia.	Prospective study	General population	Not specified	*N* = 783 Age = 70–79 years	Results indicated that 7.8% of diabetes patients had incidence of hypoglycemia, whereas 18.9% of the patients suffered from dementia. The incidence of dementia was double in patients facing hypoglycemia (*p* < 0.001). In the same way, the patients having dementia were at a higher risk of developing hypoglycemia (*p* < 0.001).
Turnbull, P.J. (2002) (UK) [29]	Assessment of nutritional status in diabetes patients and its impact on PF.	Case control study	General community	Not specified	*N* = 35 diabetes patients *N* = 35 non-diabetes patients Age > 65 years	Diabetes patients scored significantly lower on MNA (*p* < 0.01). These scores had significant correlation with BI (*p* < 0.01).
Vischer, U.M. (2010) (Switzerland) [41]	Assessment of prevalence of malnutrition elderly.	Prospective study	The Geneva Geriatric Hospital	Not specified	*N* = 146 Age > 65 years	Low scores of MNA indicated high prevalence of malnutrition in 77.1% of the diabetes patients. Moreover, in these patients, MNA scores were significantly associated with HbA1c levels (*p* = 0.0014).
Hubbard, R.E. (Canada) (2010) [35]	Comparison of prognostic value of frailty and number and severity of co-morbidities in older diabetes patients.	Longitudinal prospective cohort study	General community in five Canadian regions	Not specified	*N* = 2305 Age ≥ 70 years	There was a strong relationship between diabetes and medium-term mortality HR = 1.42 (CI 95% = 1.2–1.69). Frail diabetes patients had 2.62 times (CI 95% = 1.36–5.06) greater tendency of having diabetes complications than non-diabetes patients of same age. Moreover, the diabetes patients had more co-morbidities than non-diabetes patients (*p* < 0.005).
Maurer, M.S. (2005) (US) [17]	To investigate the association between diabetes and the risk of falls in the elderly.	Prospective cohort study	A long-term care facility	Not specified	*N* = 139 Age ≥ 60 years	The incidence rate for falls in diabetic patients as compared to non-diabetic patients was 70% and 30% respectively (*p* < 0.001).
Nelson, J.M. (2007) (US) [18]	Assessment of association between glycemic control and risk of falls in frail and non-frail elderly diabetes patients.	Retrospective, case-control study	A health maintenance organization	Not specified	*N* = 111 Age ≥ 75 years	Risk of falls increased in the patients with HbA1c levels ≤7 (*p* = 0.01).
Kalyani, R.R. (2010) (US) [19]	Assessment of the association between diabetes and functional disability in older adults, and the impact of HbA1c levels and other comorbidities on this association.	Cross-sectional, retrospective study	General community non, institutionalized population	Not specified	*N* = 6097 civilians Age ≥ 60 years	The prevalence of disability in GPA of the patients was found to be 73.6%, in LEM 52.2% and in IADL 43.6%. In addition, diabetes was associated with increased chances of disability by 2–3 times (*p* < 0.05). CVD and poor glycemic control had up to 85% more chance of diabetes-associated disabilities.
Kuo, H.K. (2005) (US) [20]	Assessment of the impact of BP and DM on physical and cognitive function.	Longitudinal prospective study	Independent living older subjects in six field sites in the US	Not specified	*N* = 2802 Age = 65–94 years	In terms of PF, patients with stage 1 (*p* = 0.03) and stage 2 (*p* = 0.007) hypertension showed a faster reduction in PF; similarly, those with DM also showed a decline in PF (*p* = 0.005), specifically in IADL. With respect to cognitive function, BP showed negative impact on memory (*p* = 0.008), stage 1 (*p* = 0.03), and stage 2 (*p* = 0.005) hypertension resulted in a reduction in reasoning; however, DM was a cause of a reduction in cognitive function DSS (*p* = 0.02).
Sinclair, A.J. (2008) (UK) [30]	Assessment of the nature of functional deterioration in older diabetes patients.	Case control study	General community	Not specified	*N* = 403 cases *N* = 403 controls Age ≥ 65 years	Diabetes patients had a greater number of comorbidities than non-diabetic patients (*p <* 0.0001) and they had a greater risk of severe functional deterioration (*p* < 0.001).
Lin, E.H. (2004) (US) [21]	Assessment of association between self-care of diabetes medication adherence, preventative services, and depression.	Cross-sectional and longitudinal retrospective survey	Primary care clinics	T2DM	*N* = 4500 Mean age of the relevant group = 63 ± 13.4 years.	Results show that 19.5% (*p* < 0.005) of the patients were non-adherent to the therapy, while 12% of the patients had major depression, which had an association with lower PF (*p* < 0.0001). Moreover, the depressed patients also had poor self-care activities (*p* < 0.0001).
Chou, K.L. & Chi, I. (1996) (China) [7]	Assessment of association between diabetes and disability, and the impact of diabetes complications on this association.	Cross-sectional study	Non-institutionalized population (general community)	Not specified	*N* = 2003 Age ≥ 60 years	Diabetic patients had a greater risk of poor performance of ADLs and IADLs than non-diabetic patients, and their inability to perform self-care was 3.5 times greater than non-diabetic patients (*p* < 0.01).
Dhamoon, M.S. (1993–2001) (US) [22]	To evaluate that diabetes acts as a long-term predictor of disability.	Prospective cohort study	General community	Not specified	*N* = 3298 Mean age of the relevant group = 69.2 years	Annual decline (*p* < 0.0001) in PF was found in the patients.
Egede, L.E. & Osborn, C.Y. (2008) (US) [23]	To evaluate the impact of depression on glycemic control and self-care.	Cross-sectional study	Internal medicine clinic	T2DM	*N* = 126 Mean age of the relevant group = 62.7 ± 11.8 years	Depression was negatively associated with social support (*p* = 0.002) and self-care activities (*p* = 0.004). Self-care of diabetes was partially associated with glycemic control (*p* = 0.08).
Gao, J. (2011) (China) [33]	To assess the impact of social support, self-efficacy, and self-care on glycemic control.	Cross-sectional study	Primary healthcare center	T2DM	*N* = 222 Age = 44–80 years	Self-care directly affected the glycemic control (*p* = 0.007); however, social support (*p* = 0.009), self-efficacy (*p* < 0.001), and PPC had an indirect effect on glycemic control.
Krein, S.L. (1998–1999) (US) [24]	Assessment of the association between chronic pain and diabetes self -management.	Cross-sectional study	Healthcare center	Not specified	*N* = 993 Age = 64 ± 10 years	Diabetes patients with chronic pain showed poor diabetes self-management and self-care (*p* = 0.002); similarly, those with severe or very severe chronic pain also reported poor self-management (*p* = 0.003) of diabetes.
Maraldi, C. (2001–2007) (US) [27]	Assessment of association between diabetes and depression.	Prospective cohort study	General community	Not specified	*N* = 2522 Age = 70–79 years	Diabetic patients had increased risk of depressed mood (*p* = 0.02) and recurrent depressed mood (*p* < 0.001) than non-diabetic patients.
Pijpers, E. (2009–2012) (Netherlands) [40]	Investigation of association between the risks of intermittent falls along with factors associated with it, and diabetes.	Longitudinal cohort study	General community	Not specified	*N* = 1145 Age ≥65 years	About 30% of the patients with diabetes had intermittent falls with an incidence rate of 129.7 per 1000 persons/year whereas, 19.4% of the subjects without diabetes had an incidence rate of intermittent falls recorded as 77.4 per 1000 persons/year HR = 1.67 (CI 95% = 1.11–2.51). Moreover, numerous physical and mental factors associated with diabetes, increased the risk of falls in diabetes patients by 47% HR = 1.3 (CI 95% = 0.79–2.11).
Schwartz, A.V. (1988–1994) (US) [25]	To assess the association between diabetes and risk of falls in older female diabetes patients.	Prospective cohort study	General community	Not specified	*N* = 9249 Age ≥ 67 years	Women with diabetes had more falls during follow-up (*p* <0.01). Diabetes and insulin use was associated with increased risk of falling among the patients i.e., more than once a year.
Sinclair, A.J. (2000) (UK) [31]	Assessment of linkage between impaired cognition self-care abilities among diabetes patients.	Case control study	General community	Not specified	*N* = 396 cases *N* = 393 controls Age ≥ 65 years	Diabetes patients having MMSE scores <23 had low levels of self-care (*p* < 0.001) and monitoring (*p* < 0.001). Association between low MMSE scores and higher hospitalization (*p* = 0.001), lower ADL (*p* < 0.001) and need of help in personal care (*p* = 0.001) was also seen.
Ulger, Z. (2002–2004) (Turkey) [38]	Assessment of malnutrition and factors associated with it in elderly.	Cross-sectional	Out-patient clinic	Not specified	*N* = 2327 Age ≥ 65 years	According to the results, 28% of the patients had poor MNA scores, which were mostly affected by depression (*p* = 0.0001), physical dependence (*p* = 0.0001), fasting plasma glucose level (*p* = 0.005), hematocrit (*p* = 0.005), ESR (*p* = 0.03), albumin (*p* = 0.002), bone mineral density (*p* = 0.007), and chronic diseases including diabetes (*p* = 0.820). The ratio of diabetes patients with and without the risk of malnutrition was 23.7%:24.2%.
Davies, M. (2006) (UK) [32]	Assessment of PDPN together with its severity and impact.	Cross-sectional descriptive study	General community	T2DM	*N* = 595 Mean age of relevant group = 67.1 ± 11.5 years	During the first phase of the study, 63.8% of the patients identified with pain. In the second phase, PDPN was found in about 19% of the patients. Furthermore, 36.8% of the patients suffered from non-neuropathic pain, and 7.4% of the patients had mixed pain. The prevalence of PDPN among the patients was 26.4%, and about 80% of those with PDPN reported moderate to severe pain, impairing their quality of life OR = 1.7 (CI 95% = 0.4–2.9%).
Galer, B.S. (1999) (US) [26]	Assessment of the nature and scope of PDN.	Cross-sectional study	Patients enrolled in a clinical trial	Not specified	*N* = 105 Age ≥ 60 years	Around 96% of the patients felt pain associated with neuropathy on their feet. Over half (53%) of the patients felt consistent pain which had become severe since the onset of PDN.
Thiel, D.M. (2011–2013) (Canada) [36]	To assess the association of compliance between physical activity recommendations and HRQoL in T2DM patients.	Prospective cohort study	Diabetes clinics, Public advertisement, primary care centers	T2DM	*N* = 1948 Mean Age = 64.5± 10.8 years	Results showed that 78.6% of the patients did not conform to the physical activity recommendations, while patients meeting the recommendations showed high scores of PF (p < 0.001), role physical (*p* = 0.001), body pain (*p* = 0.001), and physical component summary (p < 0.001) compared to the patients not meeting the required criteria.
Tabesh M. (2015) (Mauritius) [43]	Assessment of association between T2DM and physical functional disability. Moreover, determination of the degree of the association between related risk factors and diabetes.	Cross-sectional study	General community	T2DM	*N* = 3692 Mean Age = 62.1 ± 8.0	Diabetes was found to have significant association with increased risk of disability, OR = 1.76 (CI 95% = 1.34–2.08), among the study participants, having 13.2% of the prevalence of disability. Significant associations between diabetes and disability was seen among African Creoles OR = 2.03 (CI 95% = 1.16–3.56); whereas obesity highlighted the association between diabetes and disability, with an increased risk in South Asians and African Creoles of 26.3% and 12.1% respectively. The overall results showed a 67% increased risk of disability associated with diabetes.
Pai, Y.-W. (2013) (Taiwan) [42]	Assessment of the association between variation in fasting plasma glucose levels and PDPN among the T2DM patients.	Retrospective, case control study	Tertiary care hospital setting	T2DM	*N* = 2773 (enrolled) *N* = 626 (randomly selected from total) Age = 72.9 ± 10.5 years	The results showed that variation in fasting plasma glucose was significantly associated with PDPN OR = 4.08 (CI 95% = 1.60–10.42) in the third and fourth quartile, as compared to the first quartile OR = 5.49 (CI 95% = 2.14–14.06).
Yildirim, G.Z. (2014–2015) (Turkey) [39]	Assessment of nutritional status of the T2DM hospitalized patients, and highlighting the risk factors of malnutrition among such patients.	Cross-sectional study	Training and research hospital facility	T2DM	*N* = 104 Age = 65.08 ± 12.57	Results showed that the rate of malnutrition among the patients was 7.7%, whereas 18.3% patients were at risk of malnutrition. The risk factors of malnutrition among the patients were BMI <25 kg/m^2^, OR = 4.565 (CI 95% = 1.47–14.13), and duration of diabetes (15–20 years) OR = 5.535 (CI 95% = 1.15–26.6), (>20 years) OR = 7.147 (CI 95% = 1.59–31.96).
Tharek, Z. (2014–2015) (Malaysia) [44]	Assessment of the extent of self- efficacy, self-care behavior, and glycemic control and association between self-care behavior and glycemic control. Moreover, assessment of the factors associated with glycemic control among the T2DM patients.	Cross-sectional study	Primary Care Clinics	T2DM	*N* = 340 Age = 58.34 ± 11.86	Results showed the mean ± (SD) scores of self-efficacy 7.33 ± (2.25) and self-care behavior was 3.76 ± (1.87); whereas, a positive association existed between these factors r = 0.538 (*p* < 0.001). An inverse relation was found between self-efficacy and HbA1c, r = −0.41 (*p* < 0.001). Moreover, high self-efficacy has a significant association with good glycemic state, b = −0.398 (CI 95% = −0.024, −0.014), (*p* < 0.001)
Meneilly, G.S. (2015–2016) (Canada) [37]	Assessment of the status of management of T2DM of the elderly at the primary care clinics.	Cross-sectional study	Primary care clinics	T2DM	*N* = 833 Age ≥ 65 Years	Results showed that 53% participants had a HbA1c level ≤7%, the percentage of assessment for frailty, cognitive impairment, and depression was 11%, 16%, and 19% respectively; whereas, 88% and 83% assessments were of eye and foot examination respectively. Significant numbers of patients had cognitive impairment (*p* < 0.0001) and frailty (*p* < 0.0001), and a history of falls (*p* = 0.0007).
Aro, A.-K. (2015) (Finland) [45]	Assessment of HRQoL and the association between functional capability and glycemic control among the diabetes patients.	Cross-sectional study	Community-based study	Not specified	*N* = 172 Age > 65 Years	The EQ-5D scores for good glycemic control was 0.78, and for intermediate and poor glycemic control, it was 0.74 and 0.7 respectively (*p* = 0.037), HbA1c was significantly associated with poor HRQoL, r = 0.16 (CI95% = 0.01–0.31). Similarly, various domains of self-care (*p* = 0.031), mobility (*p* = 0.002), and IADL (*p* = 0.008) were compromised by poor glycemic control.
Fung, A.C.H. (2013) (China) [34]	Assessment of the association between depression and cardiac and metabolic risk factors, along with health condition among elderly T2DM patients.	Cross-sectional study	Diabetes center in a hospital setting	T2DM	*N* = 325 Age ≥ 65 Years	Depression was observed among 13% of the patients, with a positive history of co-morbidities OR = 2.84, (CI 95% = 1.35–6.00) (*p* = 0.006). The depressed patients had a longer duration of disease (mean disease duration ± (SD), 15.1 ± (9.1) versus 11.6 ± (8.1) years, (*p* = 0.02), a high frequency of hypoglycemic events (17 versus 6%) (*p* = 0.003), and poor target achievement (0 versus 16%) (*p* = 0.004).
Marden, J.R. (2006–2012) (USA) [28]	Assessment of association between diabetes, HbA1c and impaired memory among the patients with T2DM.	Prospective cohort Study Case control study (Little doubtful)	General community (noninstitutionalized population)	T2DM	*N* = 8888 Diabetics = 1837 Non Diabetics = 7051 Age = 67.4 ± 8.8	Diabetes was found to be significantly associated with a reduction of memory at a 10% faster rate (β = −0.04) per decade (CI 95% -0.06–0.01), an inverse relation was seen between HbA1c and memory loss with a 0.05 SD decline in memory score per decade (CI 95% = 0.08–0.03).

*N* = Sample size, GPA = General physical activities, LEM = lower extremity mobility, IADL = Instrumental activities of daily living, CVD = Cardiovascular diseases, PF = Physical functioning, DM = Diabetes mellitus, PB = Blood pressure, DSS = Digit symbol substitution, PPC = Patient provider communication, ADL = Activities of daily living, ESR = Erythrocyte sedimentation rate, PDPN = Painful diabetes-related peripheral neuropathy, PDN = Painful diabetes-related polyneuropathy, MNA = Mini nutritional assessment, HR = Hazard ratio, OR = Odd ratio, BI = Barthal index, CI = Confidence interval, CIB = Clock in box, CDT = Clock-drawing test, 3MS = Modified mini-mental state examination, MMSE=Mini-mental state examination, T1DM = Type 1 diabetes mellitus, DelRec = Delayed word-list recall, T2DM = Type 2 diabetes mellitus, BMI = Body mass index, HRQOL = Health-related quality of life.

**Table 3:** Quality evaluation of the included studies.

**Study Name (Reference)**	**[12]**	**[38]**	**[8]**	**[22]**	**[13]**	**[6]**	**[14]**	**[16]**	**[35]**	**[29]**
1. Question/objective sufficiently described?	2	2	2	1	2	2	2	2	2	2
2. Study design evident and appropriate?	2	2	2	2	0	1	2	2	1	2
3. Method of subject/comparison group selection or source of information/input variables described and appropriate?	2	2	2	2	2	2	2	2	2	2
4. Subject characteristics sufficiently described?	2	2	2	2	2	2	2	2	1	1
5. If interventional and random allocation was possible, was it described?	NA	NA	NA	NA	NA	NA	NA	NA	NA	1
6. If interventional and blinding of investigators was possible, was it reported?	NA	NA	NA	NA	NA	NA	NA	NA	NA	0
7. If interventional and blinding of subjects was possible, was it reported?	NA	NA	NA	NA	NA	NA	NA	NA	NA	0
8. Outcome and exposure measure(s) well-defined and robust to measurement/misclassification bias? Means of assessment reported?	2	2	2	2	2	2	2	2	2	2
9. Sample size appropriate?	2	2	2	2	1	2	2	2	2	1
10. Analytic methods described/justified and appropriate?	2	2	2	2	2	2	2	2	2	2
11. Is some estimate of variance reported for the main results?	2	2	2	2	2	2	2	2	2	2
12. Controlled for confounding factors?	2	NA	1	0	NA	NA	0	0	NA	2
13. Results reported in sufficient detail?	2	2	2	2	2	2	2	2	2	2
14. Conclusions supported by the results?	2	1	1	1	2	2	0	1	0	2
Total points	22	19	20	18	17	19	18	19	16	21
Max points possible	22	20	22	22	20	20	22	22	20	28
Summary score, in percentage	100%	95%	91%	82%	85%	95%	82%	86%	80%	75%
**Study Name (Reference)**	**[32]**	**[26]**	**[21]**	**[17]**	**[18]**	**[41]**	**[7]**	**[23]**	**[19]**	**[46]**
1. Question/objective sufficiently described?	2	2	2	2	2	1	2	2	2	2
2. Study design evident and appropriate?	2	2	0	2	2	1	2	0	2	2
3. Method of subject/comparison group selection or source of information/input variables described and appropriate?	2	2	2	2	2	2	2	2	1	1
4. Subject characteristics sufficiently described?	2	2	1	2	2	2	2	2	2	2
5. If interventional and random allocation was possible, was it described?	NA	NA	NA	NA	NA	2	NA	NA	NA	NA
6. If interventional and blinding of investigators was possible, was it reported?	NA	NA	NA	NA	NA	0	NA	NA	NA	NA
7. If interventional and blinding of subjects was possible, was it reported?	NA	NA	NA	NA	NA	0	NA	NA	NA	NA
8. Outcome and exposure measure (s) well-defined and robust to measurement/ misclassification bias? Means of assessment reported?	2	2	2	2	2	2	2	2	2	2
9. Sample size appropriate?	2	1	2	1	2	1	2	1	2	2
10. Analytic methods described/justified and appropriate?	2	0	2	2	2	2	2	2	2	2
11. Is some estimate of variance reported for the main results?	2	2	2	2	2	2	2	2	2	2
12. Controlled for confounding?	NA	NA	NA	1	1	NA	NA	NA	1	1
13. Results reported in sufficient detail?	2	2	2	2	2	2	2	1	2	2
14. Conclusions supported by the results?	2	1	2	1	1	1	2	1	2	2
Total points	20	16	17	19	20	18	20	15	20	20
Max points possible	20	20	20	22	22	26	20	20	22	22
Summary score, in percentage	100%	80%	85%	86%	91%	69%	100%	75%	91%	91%
**Study Name (Reference)**	**[33]**	**[24]**	**[27]**	**[40]**	**[25]**	**[31]**	**[15]**	**[30]**	**[20]**	
1. Question/objective sufficiently described?	2	2	2	2	2	2	2	2	2	
2. Study design evident and appropriate?	2	2	2	2	2	2	2	2	2	
3. Method of subject/comparison group selection or source of information/input variables described and appropriate?	2	2	2	2	2	2	2	2	2	
4. Subject characteristics sufficiently described?	2	2	2	2	2	2	2	2	2	
5. If interventional and random allocation was possible, was it described?	NA	NA	NA	NA	NA	0	NA	NA	NA	
6. If interventional and blinding of investigators was possible, was it reported?	NA	NA	NA	NA	NA	NA	NA	NA	NA	
7. If interventional and blinding of subjects was possible, was it reported?	NA	NA	NA	NA	NA	NA	NA	NA	NA	
8. Outcome and exposure measure (s) well-defined and robust to measurement/ misclassification bias? Means of assessment reported?	2	2	2	2	2	2	2	2	2	
9. Sample size appropriate?	1	2	2	2	2	2	2	2	2	
10. Analytic methods described/justified and appropriate?	2	2	2	2	2	2	2	1	2	
11. Is some estimate of variance reported for the main results?	2	2	2	2	2	2	2	2	2	
12. Controlled for confounding?	NA	NA	NA	NA	NA	NA	1	NA	NA	
13. Results reported in sufficient detail?	2	2	2	2	2	2	2	1	2	
14. Conclusions supported by the results?	2	2	2	1	2	1	2	2	1	
Total points	19	20	20	19	20	19	21	18	19	
Max points possible	20	20	20	20	20	22	22	20	20	
Summary score, in percentage	95%	100%	100%	95%	100%	86%	95%	90%	95%	
**Study Name (Reference)**	**[36]**	**[43]**	**[39]**	**[42]**	**[44]**	**[37]**	**[45]**	**[34]**	**[28]**	
1. Question/objective sufficiently described?	2	2	2	2	2	2	2	2	2	
2. Study design evident and appropriate?	2	2	1	2	2	2	2	2	2	
3. Method of subject/comparison group selection or source of information/input variables described and appropriate?	2	2	2	2	2	1	2	2	2	
4. Subject characteristics sufficiently described?	2	2	2	2	2	2	2	2	2	
5. If interventional and random allocation was possible, was it described?	NA	NA	NA	2	NA	NA	NA	NA	0	
6. If interventional and blinding of investigators was possible, was it reported?	NA	NA	NA	0	NA	NA	NA	NA	0	
7. If interventional and blinding of subjects was possible, was it reported?	NA	NA	NA	0	NA	NA	NA	NA	0	
8. Outcome and exposure measure (s) well-defined and robust to measurement/ misclassification bias? Means of assessment reported?	2	2	2	2	2	2	2	2	2	
9. Sample size appropriate?	2	2	1	2	2	2	1	2	2	
10. Analytic methods described/justified and appropriate?	2	2	2	2	2	2	2	2	2	
11. Is some estimate of variance reported for the main results?	2	2	1	2	2	0	2	2	2	
12. Controlled for confounding?	NA	NA	NA	2	1	NA	NA	2	2	
13. Results reported in sufficient detail?	2	2	1	2	2	2	2	2	2	
14. Conclusions supported by the results?	2	2	1	1	1	2	1	2	2	
Total points	20	20	15	23	20	17	18	22	22	
Max points possible	20	20	20	28	22	20	20	22	28	
Summary score, in percentage	100%	100%	75%	82%	90%	85%	90%	100%	78.6%	
